# ESKALE study, a French real-world study describing TRD patients with Esketamine nasal spray: final analysis

**DOI:** 10.1192/j.eurpsy.2023.271

**Published:** 2023-07-19

**Authors:** L. Samalin, L. Mekaoui, M. Rotharmel, P. de Maricourt, M.-A. Codet, E. Gaudre Wattinne

**Affiliations:** 1 Department of Psychiatry, CHU Clermont-Ferrand, University of Clermont Auvergne, Clermont-Ferrand; 2 Mental and Brain Illness Clinic, Sainte Anne Hospital GHU Paris, Paris; 3 University Department of Psychiatry, Therapeutic Centre of Excellence, Institute of Psychiatry, Rouvray Hospital Centre, Sotteville-lès-Rouen; 4 Université Paris; Department of Psychiatry, Service Hospitalo-Universitaire, GHU Paris Psychiatrie & Neurosciences, F-75014, Paris; 5 Medical Affairs department, Janssen Cilag, Issy les Moulineaux, France

## Abstract

**Introduction:**

Treatment resistant depression (TRD) affects a substantial proportion of patients with depression and carries a large unmet need. Esketamine nasal spray (NS), in combination with a selective serotonin reuptake inhibitor (SSRI) or serotonin norepinephrine reuptake inhibitor (SNRI), has been shown to reduce depressive symptoms and risk of relapse, in patients with TRD (Popova, V., et al. 2019. Am J Psychiatry; Daly, E.J., et al. 2019. JAMA Psychiatry). Esketamine NS has been authorised by European Medicines Agency as treatment for resistant depression since December 2019. ESKALE, is the first French observational study to describe TRD patients treated with Esketamine NS under real-world settings and to provide data on this innovative solution for patients.

**Objectives:**

To describe patients with TRD at Esketamine NS initiation and during the following 12-month period in real-world clinical practice.

**Methods:**

ESKALE is a French, observational, multicentre, retrospective study of adult patients with moderate to severe TRD defined as a non-response to ≥ 2 oral antidepressant. Each patient was included in one of the 3 cohorts according to Esketamine NS start date: Temporary Authorisation for Use (ATUc) cohort, post-ATU cohort or post-launch period cohort. Data were collected from medical records of patients treated with Esketamine NS between 10-29-2019 and 06-14-2022. Primary objective is to describe patients’ profile and Esketamine NS conditions of use at esketamine initiation and during the 12-month period after esketamine initiation in real-world clinical practice (either patient had stop or not the treatment). Secondary objectives are to describe Esketamine NS management, safety profile and patient pathway.

**Results:**

Two standard descriptive statistical interim analysis were conducted and published in several conferences (Samalin L, et al. Presented at EPA Hybrid congress June 2022. P.2482; Samalin L, et al. Presented at ECNP Vienna, October 2022. P.0122). This final analysis describes the data collected from medical records of patients included in the study from 04-08-2020 to 06-30-2021. 157 patients were included from 26 French centers, the majority (>65%) of patients were females. Average age was 49 years old with 27 patients > 65 years old. Duration of the current depressive episode was up to 2,5 years (mean) with an average of more than three episode in the patient’s entire life (mean). At esketamine initiation, 3 patients out of 4 were clinically perceived to have severe depression with a MADRS score of 32.0 (median). Patients had mainly depression with anxious distress specifier. Esketamine NS dose at initiation was mainly 56mg.

**Conclusions:**

Eskale is the first French cohort study generating real-world evidence on treatment resistant depression patients treated with Esketamine nasal spray. Results of the final analysis confirmed the 2 interim analysis results already published.

**Disclosure of Interest:**

None Declared

